# When Unsuspected Crystallinity Ruins Biological Testing in Early Discovery: A Case Study

**DOI:** 10.3390/ph17030284

**Published:** 2024-02-22

**Authors:** Claudi de Rocafiguera, Blanca Belsa, Mercè Font-Bardia, Cristina Puigjaner, Eduard Serra, Ana M. Cuartero-Albesa, Raimon Puig de la Bellacasa, José I. Borrell

**Affiliations:** 1 Grup de Química Farmacèutica, IQS School of Engineering, Universitat Ramon Llull, Via Augusta, 390, 08017 Barcelona, Spain; crocafiguerav@iqs.url.edu (C.d.R.); blancabelsac@iqs.url.edu (B.B.); eduard.serra@iqs.url.edu (E.S.); ana.cuartero@iqs.url.edu (A.M.C.-A.); raimon.puig@iqs.url.edu (R.P.d.l.B.); 2 Unitat de Difracció de Raigs X, Centres Científics i Tecnològics, Universitat de Barcelona, Lluís Solé i Sabarís 1-3, 08028 Barcelona, Spain; mercef@ccit.ub.edu (M.F.-B.); cris@ccit.ub.edu (C.P.)

**Keywords:** crystallinity, solubility, biological activity

## Abstract

The impact of the crystalline or amorphous structure of a solid on the solubility and pharmacokinetic properties of a drug candidate is always considered by the pharmaceutical industry during the development of a new drug; however, it is not so frequently considered during the early drug discovery process by organic and medicinal chemists, particularly those working in academia. We want to share, as an example, the false negative obtained in the biological testing of a solid sample of a tyrosine kinase inhibitor due to its unexpected crystallinity and lower solubility with respect to a solid amorphous batch of the same compound and the experimentation carried out to establish the origin of such a discrepancy.

## 1. Introduction

Pharmaceutical companies are aware of the importance of the crystalline form (or the absence of crystallinity in amorphous solids) of a drug candidate or active pharmaceutical ingredient (API) in connection with its solubility and stability and, consequently, the impact on the pharmacokinetic profile of such a product [1]. Polymorphism, defined as the ability of a molecule to form different crystal forms depending on the intermolecular associations or different conformations of the molecules in the crystal lattice, is a source of possible problems during the development of a drug (e.g., unexpected appearance of a new polymorph, disappearing polymorphs [2], or difficulties in the synthesis of the selected one) but also of opportunities from the physicochemical or patentability points of view [3].

Although the identification of the most stable polymorph is highly recommended during the early phases of drug discovery [4,5,6], it is true in our opinion that organic and medicinal chemists involved in the discovery phase usually do not consider the influence of the crystalline form of the molecules synthesized because their main objective is to have a sample for biological testing.

**Figure 1:**
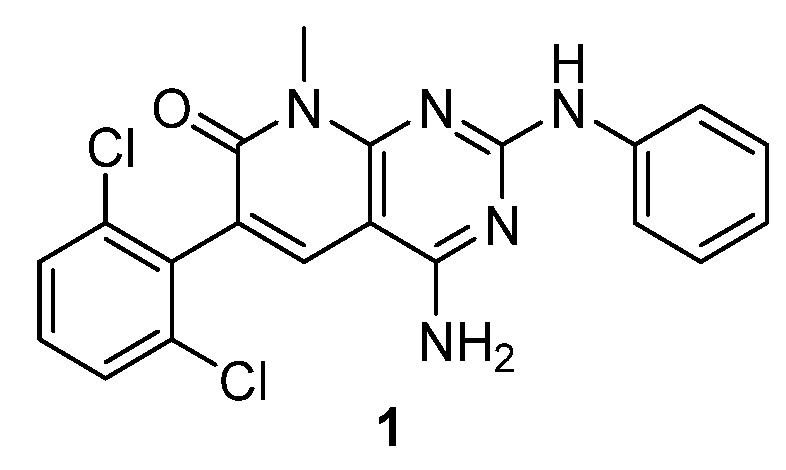
Structure of the tyrosine kinase inhibitor **1** (internally named **IQS016**).

As a part of our work in the field of tyrosine kinase inhibitors, we developed years ago compound **1** (internally named **IQS016**, Figure 1) as a candidate for the treatment of leukemia [7]. 

This compound was later licensed to the company Pangea Oncology (https://panoncology.com (accessed on 24 January 2024) and started its possible development as a DDR2 inhibitor for the treatment of squamous cell carcinoma and KRAS-mutated adenocarcinoma of the lung [8,9]. Although **1** presented very good in vitro activity, the development was stopped by Pangea Oncology due to the poor pharmacokinetic properties. Nevertheless, a preliminary GMP batch of **1** (named **PB1** by Pangea Oncology) was prepared by Applus^+^ Laboratories (https://www.appluslaboratories.com (accessed on 24 January 2024), a sample of which was transferred to our laboratory. Very recently and in connection with a project in the field of pancreatic cancer, we sent two solid samples of compound **1** for the determination of the in vitro inhibitory capability of the most relevant tyrosine kinases involved in such a disease to Reaction Biology (https://www.reactionbiology.com (accessed on 24 January 2024). One of them came from a batch of compound **1** obtained at our laboratories in 2014 and stored at room temperature (named **IQS016**) and the other from the same compound prepared by Applus^+^ Laboratories (named **PB1**). Although both samples seemed indistinguishable regarding their appearance as powdery solids and were prepared using the same synthetic route [7], the contradictory results obtained (the **IQS016** sample being active in front of several of the kinases studied but the **PB1** sample being totally inactive) forced us to perform the complementary research that is described in this paper.

## 2. Results and Discussion

**Figure 2:**
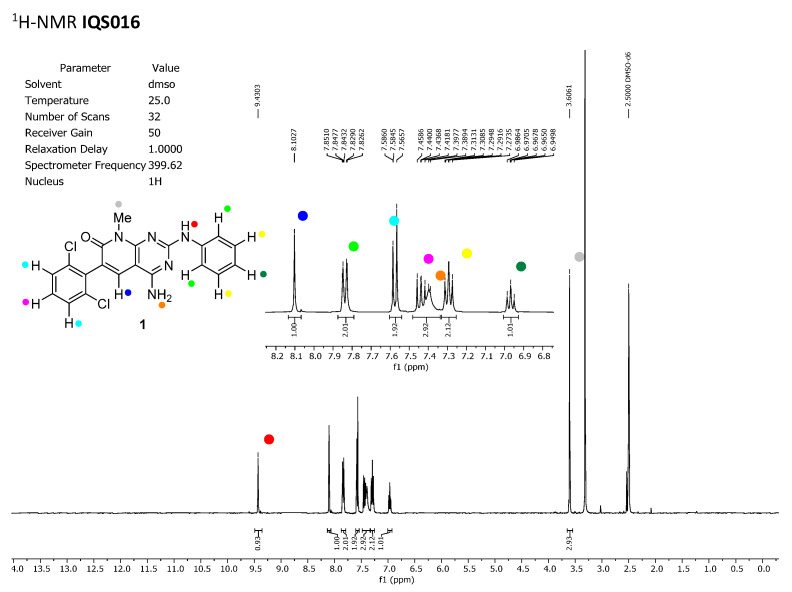
^1^H-NMR spectrum of **IQS016** recorded in DMSO-*d*_6._

**Figure 3:**
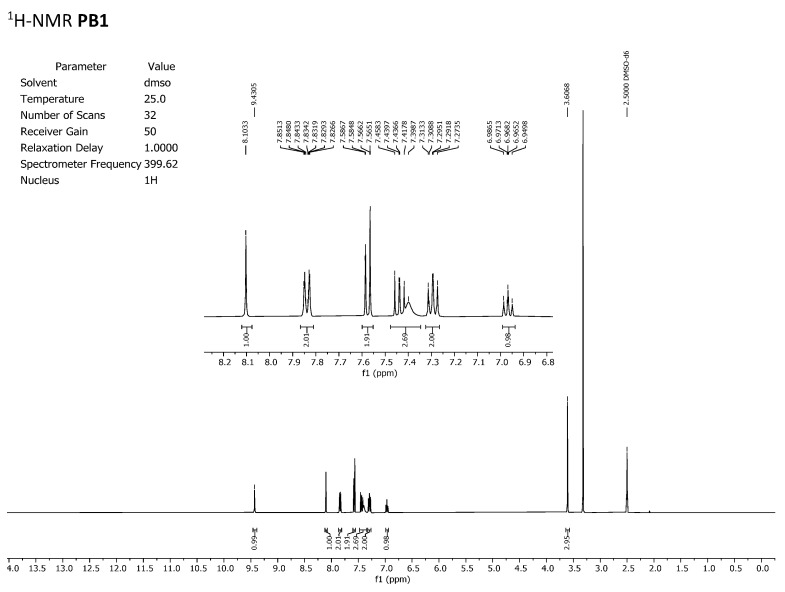
^1^H-NMR spectrum of **PB1** recorded in DMSO-*d_6._*

To discard any possible structural differences between the samples **IQS016** and **PB1** or the presence of an impurity in any of the samples that could justify the differences observed in the in vitro tests with isolated receptors, we first recorded the ^1^H-NMR spectrum of each sample. The spectra recorded in DMSO-*d_6_* are included in Figure 2 and Figure 3 and clearly show that the spectra are superimposable, confirming that the structure of the molecule present in both samples is the same.

**Figure 4:**
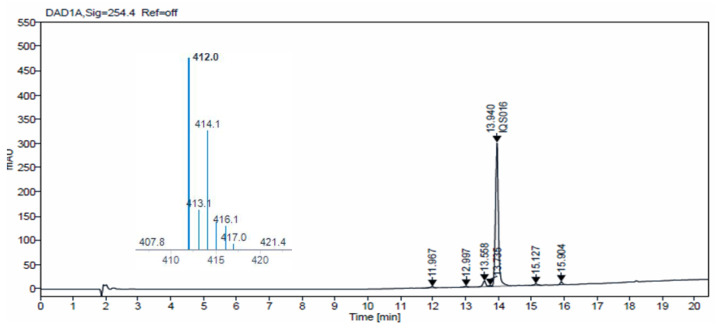
Chromatogram obtained for **IQS016** at 254 nm and mass spectrum of the peak at a retention time of 13.94 min in positive mode [M + H]^+^ 412.

**Figure 5:**
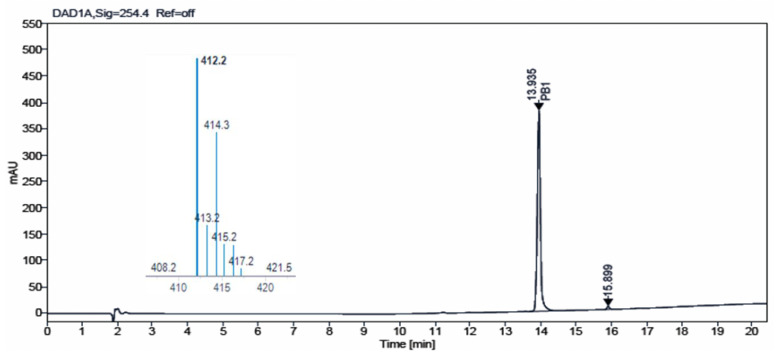
Chromatogram obtained for **PB1** at 254 nm and mass spectrum of the peak at a retention time of 13.94 min in positive mode [M + H]^+^ 412.

Additionally, we carried out HPLC-MS analysis of both samples. Such analysis was performed using an HPLC-MS, Agilent Technologies 1200 series LC/LC MSD iQ, column X-bridge C18 (100 × 4.6 × 3.5 µm, waters) at oven temperature 40 °C and a combined isocratic and linear gradient elution at a flow rate of 0.5 mL min^−1^ consisting of a mobile phase of water and acetonitrile, each containing 0.1% formic acid (*v*/*v*), over a 20 min run time. Detection was performed at 254 nm and by the MS ionization method with cone voltage 110 V and MS scan 100–1000. Figure 4 and Figure 5 show the chromatograms obtained for **IQS016** and **PB1** at 254 nm. Integration by normalized areas reveals a purity of 91.5% for **IQS016** and 99.8% for **PB1**. In both cases, the retention time of the main peak is 13.94 min. Each one of the chromatograms shows the mass spectrum of the main peak recorded using electrospray ionization (ESI) in the positive mode (*m*/*z* = 412, M^+^ + 1), revealing the presence of two chlorine atoms in the molecule.

Once we had demonstrated that both samples, **IQS016** and **PB1**, contain the same molecular structure and that the presence of unknown impurities does not justify the difference observed in the biological testing, although the appearance of both solid samples was almost the same (a slightly colored powder), we decided to evaluate the possible differences in the crystallinity of both solids.

Differential Scanning Calorimetry (DSC) is a good method to discern the presence in a solid of different crystalline forms of the same chemical substance [10,11,12]. As is already known, different crystalline forms exhibit different melting points, and if the solid is amorphous, it does not have a well-defined melting point. However, its glass transition and subsequent crystallization can be measured. If the concentration of one of the crystalline forms in the sample is very low (less than 5%), two melting peaks are not detected. Instead, a broader single peak is observed.

**Figure 6:**
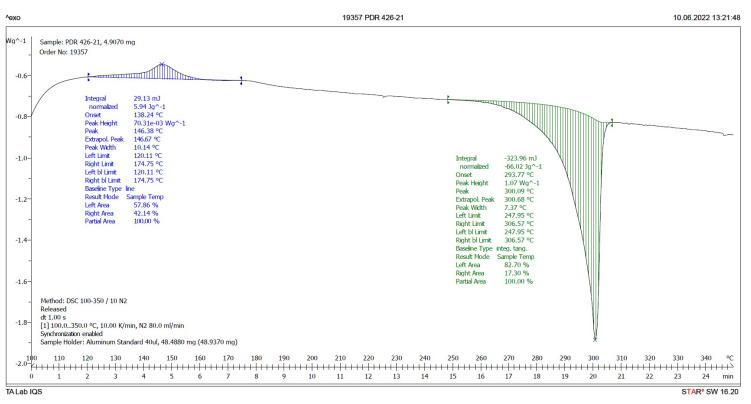
DSC plot of **IQS016**.

**Figure 7:**
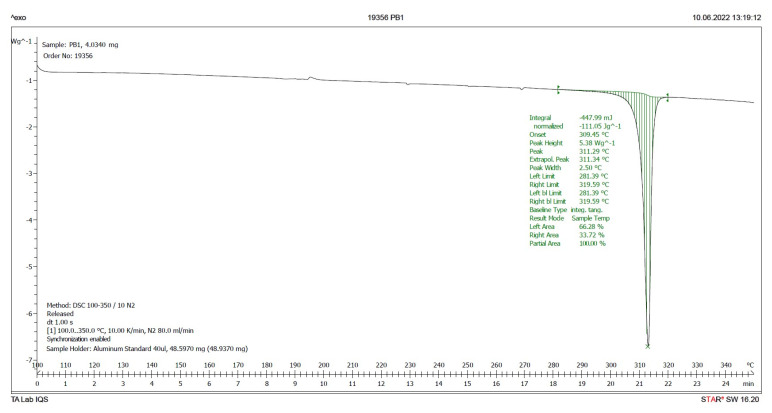
DSC plot of **PB1**.

Therefore, a DSC analysis of samples **IQS016** and **PB1** was carried out. Figure 6 and Figure 7 present the evaluated records corresponding to these different samples.

The main results derived from the evaluated DSC plots are presented in Table 1.

There is a difference of almost 16 degrees between the onset values and 11 degrees between the peak temperature values of the **IQS016** and **PB1** samples. Consequently, considering that they are chemically identical substances, the two records can only correspond to two different solid forms of the same substance.

**Figure 8:**
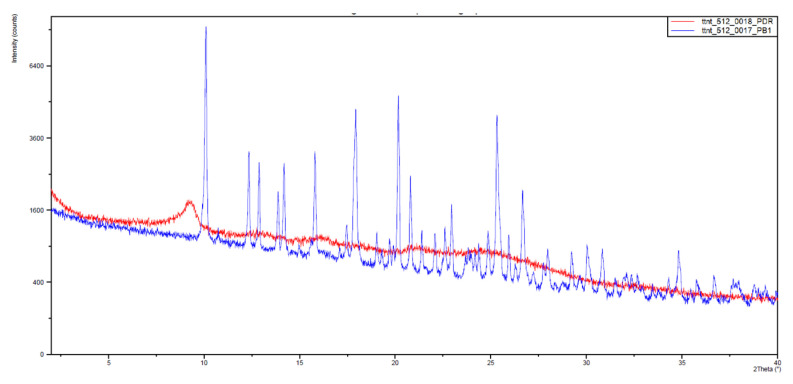
Powder X-ray diffractograms of **PB1** (blue) and **IQS016** (red) from 2 to 40 2θ.

With this, somehow surprising, result in hand, we decided to measure the X-ray powder diffraction diagrams of both samples that are depicted in Figure 8. The **IQS016** sample (Figure 8, red) is mainly not microcrystalline, containing amorphous/partially crystalline/nanocrystalline phases. On the contrary, the **PB1** sample (Figure 8, blue) is microcrystalline, as its X-ray powder diffractogram shows. That is to say, although both powders look quite similar, **PB1** presents a crystalline structure that could justify a lower solubility in organic solvents and water than **IQS016**.

**Figure 9:**
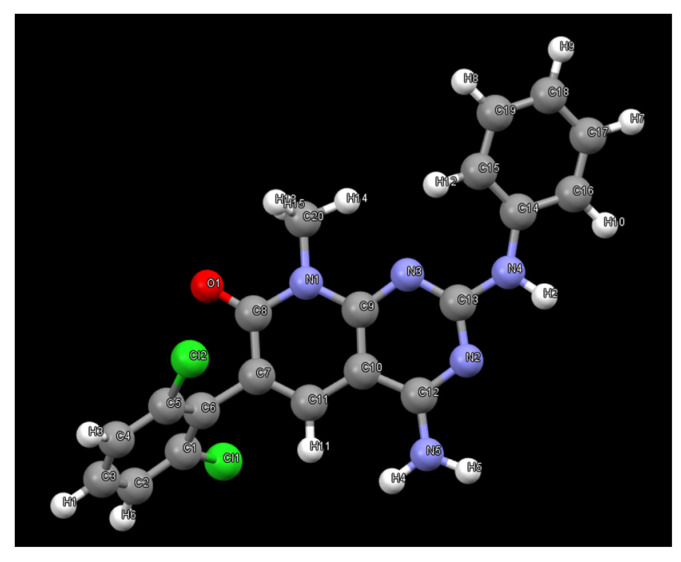
Structure of **1** obtained from X-ray powder diffraction data of **PB1**.

Subsequently, we decided to try to carry out the determination of the structure present in **PB1** from the X-ray powder diffraction data [13] to confirm that such crystals contain compound **1**. The powder X-ray diffractogram of **PB1** was perfectly indexed to an orthorhombic unit cell with unit cell parameters a = 18.63 Å, b = 17.49 Å and c = 12.46 Å and a volume of 4062.6 Å^3^. The number of molecules in the unit cell was calculated to be Z = 8. The space group *Pbca* was assigned based on the systematic absences and the subsequent Pawley pattern matching [14] fitted very well with the experimental X-ray powder diffractogram, with an agreement factor of 2.63%. Its crystal structure was solved using the Global Optimization Simulated Annealing approach integrated into Topas [15,16]. Some constraints were introduced, considering the molecule as a rigid body using the Z-matrix notation, which was allowed to rotate and translate in the three directions within the unit cell. Planar restrictions were applied to the aromatic rings and the phenyl and dichlorophenyl rings were allowed to rotate about two fixed points. A chemical sense solution with an agreement factor of 13.4% was obtained. The crystal structure so obtained was subsequently refined by the Rietveld method, also using TOPAS v6 software, giving a satisfying result with a low Rwp value of 6.59%. The final pattern matching and Rietveld plots for the crystal structure refinement are shown in the supporting information. Figure 9 shows the structure present in the **PB1** sample determined from the powder X-ray diffraction data. As can be seen, the structure corresponds to the tyrosine kinase inhibitor **1** (Figure 1).

Convergently, we decided to try to obtain single crystals from a sample of **IQS016**, which is more soluble than **PB1**, using the evaporative crystallization technique. Approximately 4 mg of the **IQS016** sample was dissolved in MeOH, acetone, and DMSO. Two replicates of each sample, one in a closed vial and the other open to the air, were prepared. After one day, crystals appeared in the open samples in MeOH and acetone, and after one week, in the open sample in DMSO. In no case were crystals formed in the closed vials.

**Figure 10:**
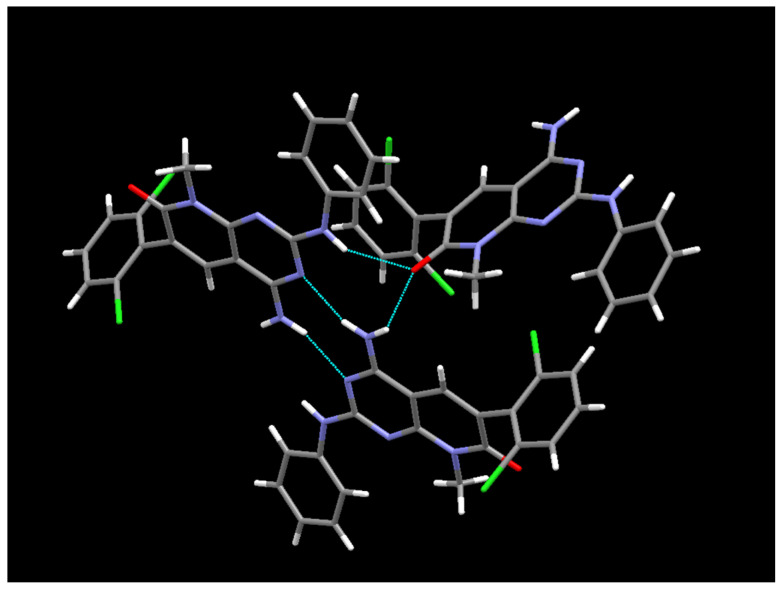
Hydrogen bonds formed between three **1** molecules in the crystal grown in MeOH.

The crystal structure present in the crystals grown in MeOH and DMSO from the **IQS016** sample was determined by single-crystal X-ray diffraction. Here, **1** crystallizes from MeOH in the orthorhombic space group *Pbca*, with Z = 8 for the formula unit C_20_H_15_Cl_2_N_5_O. The ORTEP diagram and atomic numbering, together with the crystallographic data, are summarized in the supporting information. This structure is the same anhydrous form as the one obtained from the powder X-ray diffraction data; however, in this case, it has been determined at 100 K. The structure presents an arrangement of three hydrogen bonds between three molecules of **1** (Figure 10), forming an R^2^_3_(8) supramolecular heterosynthon [17]: between the C7 carbonyl group and N-H at C2 (2.27 Å), between the same carbonyl group and one of the hydrogens of the NH_2_ group at C4 (2.14 Å), and between the second hydrogen of such NH_2_ group at C4 and the pyrimidine ring nitrogen N3 (2.07 Å).

**Figure 11:**
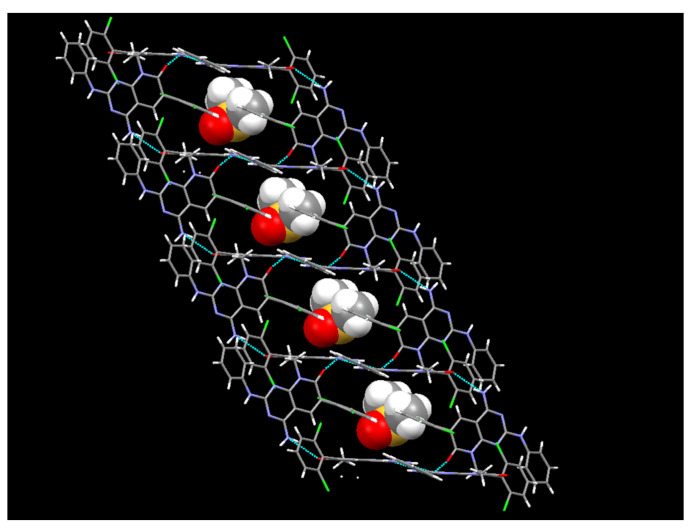
The crystalline structure of the DMSO solvate of **1**.

In the case of the crystals grown in DMSO, the single crystal X-ray diffraction analysis showed that compound **1** crystallizes in the monoclinic space group *C12/c1*, with Z = 8 for the formula unit C_22_H_21_Cl_2_N_5_O_2_S, thus confirming a 1:1 co-crystallization between **1** and DMSO. The ORTEP diagram and atomic numbering, together with the crystallographic data, are summarized in the supporting information. In such a solvate, the DMSO, being one of the best hydrogen-bond acceptors, is not involved in any hydrogen bond with **1** and occupies the spaces between the **1** molecules (Figure 11). In fact, the hydrogen bonds between the pyridopyrimidine molecules are the same as those present in the crystals grown from MeOH.

The different crystalline natures of the samples of the tyrosine kinase inhibitor **1** named **IQS016** and **PB1**, the first amorphous and the second microcrystalline, can be the origin of the inconsistency observed in the results of the biological testing of both samples carried out at Reaction Biology. The test consists of a radiometric protein kinase assay (33PanQinase^®^ Activity Assay) used for measuring the kinase inhibitory activity of the samples against the selected isolated kinases. The results are expressed as the residual activity of the selected kinases after treatment with the indicated compounds at a certain µM concentration. Staurosporine is used as a positive control.

Reaction Biology offers two options for compound preparation and shipping to their facilities in Germany: as frozen DMSO stock solutions packed in dry ice or as solids. During our research in the field of tyrosine kinase inhibitors, we have routinely sent the compounds as solids but, probably, the crystalline and less soluble form of **PB1** has affected its results.

To prove such a hypothesis, we sent for evaluation three different samples: one solid sample of **IQS016**, one solid sample of **PB1**, and a sample of **PB1** dissolved in DMSO and frozen. For comparison purposes, we selected four tyrosine kinases of the whole set considered in our study on pancreatic cancer: EGFR (Epidermal Growth Factor Receptor), FGFR1 (Fibroblast Growth Factor Receptor 1), FGFR2 (Fibroblast Growth Factor Receptor 2), and VEGFR2 (Vascular Endothelial Growth Factor Receptor-2). The **PB1** samples were evaluated at two different concentrations, 0.5 and 10 µM, while the **IQS016** sample used as a reference was evaluated at 10 µM. The results obtained are summarized in Table 2.

As can be seen in the table, while the solid sample of **PB1** is almost inactive (only presents an intermediate activity for EGFR at 10 µM, which is the kinase most sensible to compound **1**), the activity of the **PB1** sample sent dissolved in DMSO presents the same activity profile and similar residual activity values at 10 µM as those of the **IQS016** sent as a solid. So, we can conclude that the false negative obtained for the activity values of the **PB1** sample observed and described at the beginning of this paper was due to the unexpected crystallinity and lower solubility of the **PB1** sample.

Finally, concerning the origin of the crystallinity of the **PB1** sample, we must exclude that this is due to a different synthetic itinerary because both samples, **IQS016** and **PB1**, were prepared using the same protocol described by our group [7]. However, Applus^+^ Laboratories introduced a difference in the final purification because while the **IQS016** was purified using column chromatography and concentrated in vacuo to afford the corresponding solid, the **PB1** was suspended in acetone at reflux and stirred for 30 min and then cooled at room temperature and filtered. Without any doubt, this disaggregation was responsible for the crystallinity of such a sample.

We consider that this example case is a good warning for organic and medicinal chemists working in the discovery phase of the possible impact of crystallinity and polymorphism during a phase of the research of a new drug in which such solid-state properties are not routinely considered. In our case, this experience has convinced us to send the compounds to Reaction Biology in a DMSO solution from now on.

## 3. Materials and Methods

### 3.1. HPLC-MS Study of the **IQS016** and **PB1** Samples

Chemicals and reagents:

The reagents used are, acetonitrile (ACN) of HPLC-MS grade (83640.320) from VWR, HPLC-MS grade formic acid optima (A117-60) from Fisher Chemical, and dimethyl sulfoxide (DMSO) LC-MS grade (85190) from Thermo Scientific, and ultra-purified water was prepared using a Milli-Q purification system (Milli-Q Integral 3, Millipore).

Samples:

**IQS016** was synthesized at IQS by the *Grup de Química Farmacèutica.* **PB1** was obtained from APPlus. The working sample solutions were prepared by dissolving an accurately weighed 1.5 mg of each sample in 1.5 mL of DMSO. The sample solutions were obtained from the dilution of 70 µL of working sample solutions in 1.5 mL of ACN (0.05 mg/mL). The volume was filtered through a 0.45 μm nylon syringe filter (FILTER-LAB JNY045025N).

Equipment, column, experimental conditions:

The liquid chromatography analysis was performed using an HPLC-MS, Agilent Technologies 1200 series LC/LC MSD iQ, column X-bridge C18 (100 × 4.6 × 3.5 µm, waters) and a combined isocratic and linear gradient mode of elution (Table 3) at a flow rate of 0.5 mL min^−1^, consisting of a mobile phase of water (A) and acetonitrile (B), each containing 0.1% formic acid (*v*/*v*), over a 20 min run time. The oven temperature was 40 °C and the sample injection volume was set at 10 μL.

The ESI-MS interface was operated in the positive ionization mode at gas temperature 350 °C, gas flow 10 L/min., nebulizer 45 psi, and capillary voltage 4000 V. 

Detection was performed at 254 nm, ionization method with cone voltage 110 V and MS scan 100–1000, SIM (*m*/*z*) 412, positive mode, 410 negative mode. Figures S1–S4 in the Supplementary Materials include the chromatograms and mass spectra of the samples **IQS016** and **PB1**.

### 3.2. X-ray Powder Diffraction Analysis of Samples **IQS016** and **PB1**

Sample preparation:

Parts of the submitted powder materials were sandwiched between films of polyester of 3.6 microns of thickness.

Instrument and experimental conditions:

*PANalytical X’Pert PRO MPD* θ/θ powder diffractometer of 240 mm of radius in a configuration of convergent beam with a focalizing mirror and a transmission geometry with flat samples sandwiched between low absorbing films.

Cu Kα radiation (λ = 1.5418 Å).
Work power: 45 kV–40 mA.
Incident beam slits defining a beam height of 0.4 mm.
Incident and diffracted beam 0.02 radians *Soller* slits
*PIXcel* detector: Active length = 3.347°.

Then, 2θ scans from 2 to 60 °2θ with a step size of 0.026 °2θ and a measuring time of 300 s per step.

Table S2 in the Supplementary Information includes the peak list for the **PB1** sample. Figures S5 and S6 show the X-ray powder diffraction diagrams of the samples **IQS016** and **PB1**, respectively.

### 3.3. Determination of the Crystal Structure of **PB1** from X-ray Powder Diffraction Data

The powder X-ray diffraction pattern was obtained on a PANalytical X’Pert PRO MPD diffractometer of 240 mm in radius in a transmission configuration with a spinner glass capillary sample holder, using Cu K_α1+2_ radiation (λ = 1.5418 Å), with a focalizing elliptic mirror and a PIXcel detector working at a maximum detector’s active length of 3.347°. Incident and diffracted beam 0.02 radians soller slits and incident beam slits defining a beam height of 0.4 mm have been used with the sample placed in a glass capillary. Ten consecutive 2theta/theta scans were measured and added from 2 to 70° in 2θ, with a step size of 0.013° and a measuring time of 300 s per step (total measuring time 18 h).

The elucidation of the crystal structure was attempted from the powder X-ray diffraction data. The powder X-ray diffractogram of the **PB1** was perfectly indexed to an orthorhombic unit cell with unit cell parameters a = 18.63 Å, b = 17.49 Å and c = 12.46 Å and a volume of 4062.6 Å^3^. Taking into account the unit cell volume, the molecular weight of the compound and an estimated density value of 1.2 Mg/m^3^, the number of molecules in the unit cell was calculated to be Z = 8. The space group *Pbca* was assigned based on the systematic absences and the subsequent Pawley pattern matching fitted very well the experimental X-ray powder diffractogram, being an agreement factor of 2.63%. Its crystal structure was solved using the Global Optimization Simulated Annealing approach integrated in Topas and the crystal structure of the DMSO solvate of the **IQS016** (see Section 3.5) was used as a starting model. Some constraints were introduced, considering the molecule as a rigid body using the Z-matrix notation, which was allowed to rotate and translate in the three directions within the unit cell. Planar restrictions were applied to the aromatic rings and the phenyl and dichlorophenyl rings were allowed to rotate about two fixed points. A chemical sense solution with an agreement factor of 13.4% was obtained. The crystal structure so obtained was subsequently refined by the Rietveld method, also by means of TOPAS v6 software, giving a satisfying result with low Rwp value of 6.59%. Figures S7 and S8 and Table S3 in the Supplementary Materials include the pattern matching Pawley fit plot of **1**, final Rietveld plot for the crystal structure refinement of **1**, and the most relevant parameters of the crystal structure determination and refinement of **PB1**, respectively.

### 3.4. Crystal Structure Determination of a Single Crystal of **IQS016** Grown in MeOH

A colorless prism-like specimen of C_20_H_15_Cl_2_N_5_O, approximate dimensions 0.104 mm × 0.129 mm × 0.413 mm, was used for the X-ray crystallographic analysis. The X-ray intensity data were measured on a D8 Venture system equipped with a multilayer monochromator and a Mo microfocus (λ = 0.71073 Å).

The frames were integrated with the Bruker SAINT software package (version V8.40B) using a narrow-frame algorithm. The integration of the data using an orthorhombic unit cell yielded a total of 79,253 reflections to a maximum θ angle of 26.40° (0.80 Å resolution), of which 4037 were independent (average redundancy 19.632, completeness = 99.9%, Rint = 5.16%, Rsig = 1.49%) and 3596 (89.08%) were greater than 2σ(F2). The final cell constants of a = 16.9088(6) Å, b = 12.6026(4) Å, c = 18.5117(5) Å, and volume = 3944.7(2) Å^3^ are based upon the refinement of the XYZ-centroids of reflections above 20 σ(I). Data were corrected for absorption effects using the multi-scan method (SADABS). The calculated minimum and maximum transmission coefficients (based on crystal size) are 0.7161 and 0.7454. 

The structure was solved and refined using the Bruker SHELXTL Software Package (version 2019/1) [18], using the space group Pbca, with Z = 8 for the formula unit, C_20_H_15_Cl_2_N_5_O. The final anisotropic full-matrix least-squares refinement [19] on F2 with 253 variables converged at R1 = 4.63% for the observed data and wR2 = 10.94% for all the data. The goodness-of-fit was 1.149. The largest peak in the final difference electron density synthesis was 0.424 e-/Å^3^ and the largest hole was −0.448 e-/Å^3^, with an RMS deviation of 0.063 e-/Å^3^. On the basis of the final model, the calculated density was 1.388 g/cm^3^ and F(000), 1696 e-. Figure S9 in the Supplementary Materials shows the ORTEP diagram and atomic numbering of **1**. Tables S4 to S10 include the crystal data for structure **1** (mo_023VB113_0ma_a).

### 3.5. Crystal Structure Determination of a Single Crystal of **IQS016** Grown in DMSO

A colorless prism-like specimen of C_22_H_21_Cl_2_N_5_O_2_S, approximate dimensions 0.092 mm × 0.123 mm × 0.266 mm, was used for the X-ray crystallographic analysis. The X-ray intensity data were measured on a D8 Venture system equipped with a multilayer monochromator and a Mo microfocus (λ = 0.71073 Å).

The frames were integrated with the Bruker SAINT software package using a narrow-frame algorithm. The integration of the data using a monoclinic unit cell yielded a total of 34,473 reflections to a maximum θ angle of 27.15° (0.78 Å resolution), of which 4959 were independent (average redundancy 6.952, completeness = 99.6%, Rint = 3.69%, Rsig = 2.15%) and 4190 (84.49%) were greater than 2σ(F2). The final cell constants of a = 20.0528(9) Å, b = 11.8577(5) Å, c = 18.8759(8) Å, β = 90.855(2)°, and volume = 4487.8(3) Å^3^ are based upon the refinement of the XYZ-centroids of reflections above 20 σ(I). Data were corrected for absorption effects using the multi-scan method (SADABS). The calculated minimum and maximum transmission coefficients (based on crystal size) are 0.7026 and 0.7455. 

The structure was solved and refined using the Bruker SHELXTL Software Package, using the space group C12/c1, with Z = 8 for the formula unit, C_22_H_21_Cl_2_N_5_O_2_S. The final anisotropic full-matrix least-squares refinement on F2 with 330 variables converged at R1 = 3.25% for the observed data and wR2 = 8.54% for all the data. The goodness-of-fit was 1.067. The largest peak in the final difference electron density synthesis was 0.396 e-/Å^3^ and the largest hole was −0.322 e-/Å^3^, with an RMS deviation of 0.056 e-/Å^3^. On the basis of the final model, the calculated density was 1.452 g/cm^3^ and F(000), 2024 e-. Figure S10 in the Supplementary Materials shows the ORTEP diagram and atomic numbering of a DMSO solvate of **1**. Tables S11 to S14 include the crystal data for a DMSO solvate of **1** (mo_023VB102_0m_a).

### 3.6. Selectivity Profiling of Samples **IQS016** and **PB1** Using 4 Protein Kinases

A radiometric protein kinase assay (33PanQinase^®^ Activity Assay) was used for measuring the kinase activity of the 4 protein kinases (EGFR, FGFR1, FGFR2, and VEGFR2). All the kinase assays were performed in 96-well ScintiPlates^TM^ from Perkin Elmer (Boston, MA, USA) in a 50 µL reaction volume. The reaction cocktail was pipetted in 4 steps in the following order:

- 10 µL of non-radioactive ATP solution (in H2O)
- 25 µL of assay buffer/[γ-33P]-ATP mixture
- 5 µL of test sample in 10% DMSO
- 10 µL of enzyme/substrate mixture

The assay for all the protein kinases contained 70 mM HEPES-NaOH pH 7.5, 3 mM MgCl2, 3 mM MnCl_2_, 3 µM Na-orthovanadate, 1.2 mM DTT, 50 μg/mL PEG20000, ATP (variable concentrations, corresponding to the apparent ATP-Km of the respective kinase), [γ-33P]-ATP (approx. 8 × 1005 cpm per well), protein kinase (variable amounts), and substrate (variable amounts). 

All the protein kinases provided by RBE were expressed in Sf9 insect cells or in *E. coli* as recombinant GST-fusion proteins or His-tagged proteins, either as full-length or enzymatically active fragments. All the kinases were produced from human cDNAs. The kinases were purified by affinity chromatography using either GSH-agarose or immobilized metal. Affinity tags were removed from a number of kinases during purification. The purity of the protein kinases was examined by SDS-PAGE/Coomassie staining. The identity of the protein kinases was checked by mass spectroscopy. 

Kinases from external vendors (CAR = Carna Biosciences Inc.; INV = Life Technologies (Invitrogen Corporation); MIL = Merck-Millipore (Millipore Corporation)) were expressed, purified, and quality-controlled by virtue of the vendors’ readings.

The reaction cocktails were incubated at 30 °C for 60 min. The reaction was stopped with 50 µL of 2% (*v*/*v*) H_3_PO_4_, and the plates were aspirated and washed two times with 200 µL 0.9% (*w*/*v*) NaCl. The incorporation of 33Pi (counting of “cpm”) was determined with a microplate scintillation counter (Microbeta, Wallac).

For each kinase, the median value of the cpm of three wells with complete reaction cocktails, but without kinase, was defined as “low control” (n = 3). This value reflects the unspecific binding of radioactivity to the plate in the absence of protein kinase but in the presence of the substrate. Additionally, for each kinase, the median value of the cpm of three other wells with the complete reaction cocktail, but without any compound, was taken as the “high control”, i.e., full activity in the absence of any inhibitor (n = 3). The difference between the high and low control was taken as 100% activity for each kinase. 

As part of the data evaluation, the low control value of each kinase was subtracted from the high control value as well as from the corresponding “compound values”. The residual activity (in %) for each compound well was calculated using the following formula:

Res. Activity (%) = 100 × [(cpm of compound − low control)/(high control − low control)]

As a parameter for assay quality, the Z’-factor [20] for the low and high controls of each assay plate (n = 8) was used. RBE’s criterion for the repetition of an assay is a Z’-factor below 0.4 [21]. The results obtained are included in Table 2.

## Figures and Tables

**Table 1:** Results of the endotherm phenomena from the evaluated DSC plots of **IQS016** and **PB1** ^1^.

Sample	Onset (°C)	Peak (°C) ^2^	delta H (J/g)	Peak Height (W/g)
**IQS016**	293.8	300.1	66.02	1.07
**PB1**	309.5	311.3	111.05	5.38

^1^ The experimental conditions used to carry out the DSC runs are **IQS016** (4.907 mg), **PB1** (4.034 mg), and the aluminum crucible from 100 to 350 °C at 10 K/min in a Mettler Toledo DSC821. ^2^ The melting point is determined by the temperature value referred to as the onset.

**Table 2:** The kinase inhibition profile of **IQS016, PB1**, and **PB1** dissolved in DMSO was determined by measuring the residual activity values at two concentrations in 4 protein kinase assays ^1^.

Sample	PB1		PB1 (DMSO)		IQS016	Staurosporine
**Conc. (µM)**	**0.5**	**10**	**0.5**	**10**	**10**	**10**
EGFR	56	43	23	12	16	8
FGFR1	96	89	54	30	21	0
FGFR2	99	91	47	19	15	0
VEGFR2	103	94	88	40	33	0

^1^ Residual activities (% of control), residual activity ≤ 50%, residual activity ≤ 20%.

**Table 3:** Chromatographic gradient.

Time (min)	Mobile Phase A (*v*/*v*)	Mobile Phase B (*v*/*v*)
0.0–5.0	70	30
15.0	0	100
15.0–20.0	0	100

## Data Availability

Data are contained within the article or supplementary material. CCDC 2325663 and 2325664 contain the supplementary crystallographic data for this paper. These data can be obtained free of charge from the Cambridge Crystallographic Data Centre via www.ccdc.cam.ac.uk/structures (accessed on 24 January 2024).

## Supplementary Materials

The following supporting information can be downloaded at https://www.mdpi.com/article/10.3390/ph17030284/s1, HPLC-MS study of the IQS016 and PB1 samples; X-ray powder diffraction analysis of IQS016 and PB1; Determination of the crystal structure of PB1 from the X-ray powder diffraction data; Crystal structure determination of a single crystal of IQS016 grown in MeOH; Crystal structure determination of a single crystal of IQS016 grown in DMSO.
