# Supplementary material for: When Unsuspected Crystallinity Ruins Biological Testing in Early Discovery: A Case Study
Source: Pharmaceuticals (Basel). 2024 Feb 22;17(3):284. doi: 10.3390/ph17030284 (PMC10976151; doi:10.3390/ph17030284)
Supplement: Supplementary file 1 [file pharmaceuticals-17-00284-s001.zip › pharmaceuticals-2866158-supplementary.pdf]

SUPPLEMENTARY INFORMATION

When Unsuspected Crystallinity Ruins Biological Testing in Early Discovery: A Case Study

Claudi de Rocafiguera [1], Blanca Belsa [1], Mercè Font-Bardia [2], Cristina Puigjaner [2], Eduard Serra [1], Ana M. Cuartero-Albesa [1], Raimon Puig de la Bellacasa [1] and José I. Borrell [1,*]

[1] Grup de Química Farmacèutica, IQS School of Engineering, Universitat Ramon Llull, Via Augusta, 390, 08017 Barcelona, Spain
[2] Unitat de Difracció de Raigs X, Centres Científics i Tecnològics, Universitat de Barcelona, Lluís Solé i Sabarís 1-3, 08028 Barcelona, Spain
* Correspondence: jose.borrell@iqs.url.edu

Table of Contents

	Page
1.1 HPLC-MS study of the IQS016 and PB1 samples	2
1.2 X-ray powder diffraction analysis of IQS016 and PB1	5
1.3 Determination of the crystal structure of PB1 from X-ray powder diffraction data	7
1.4 Crystal structure determination of a single crystal of IQS016 grown in MeOH	9
1.5 Crystal structure determination of a single crystal of IQS016 grown in DMSO	19

Experimental Procedures

1.1. HPLC-MS study of the IQS016 and PB1 samples

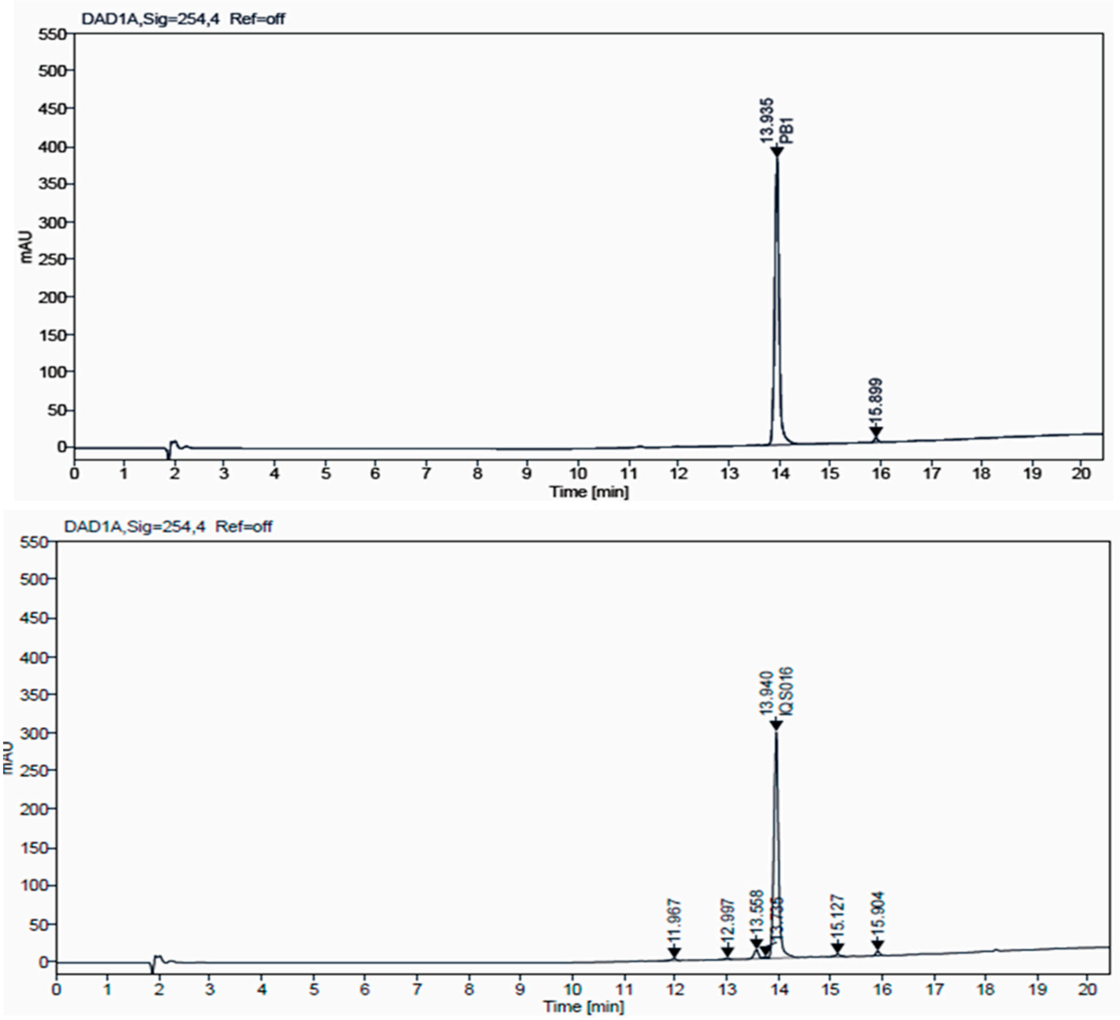

Figure S1: Chromatograms at 254 nm of **PB1** and **IQS016**

Figure S2 shows the UV profile of both compounds, with both samples displaying the same profile.

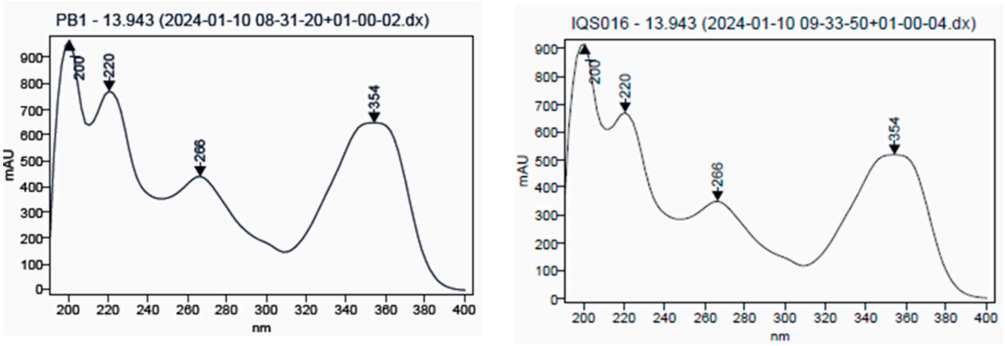

Figure S2: PB1 and IQS016 UV profile

Figures S3 and S4, display the mass spectrum positive of peak retention time 13,94 min $[M+H]^+$ 412, for samples PB1 and IQS016 respectively. Both samples present the same profile an fulfill with sample mass 411.

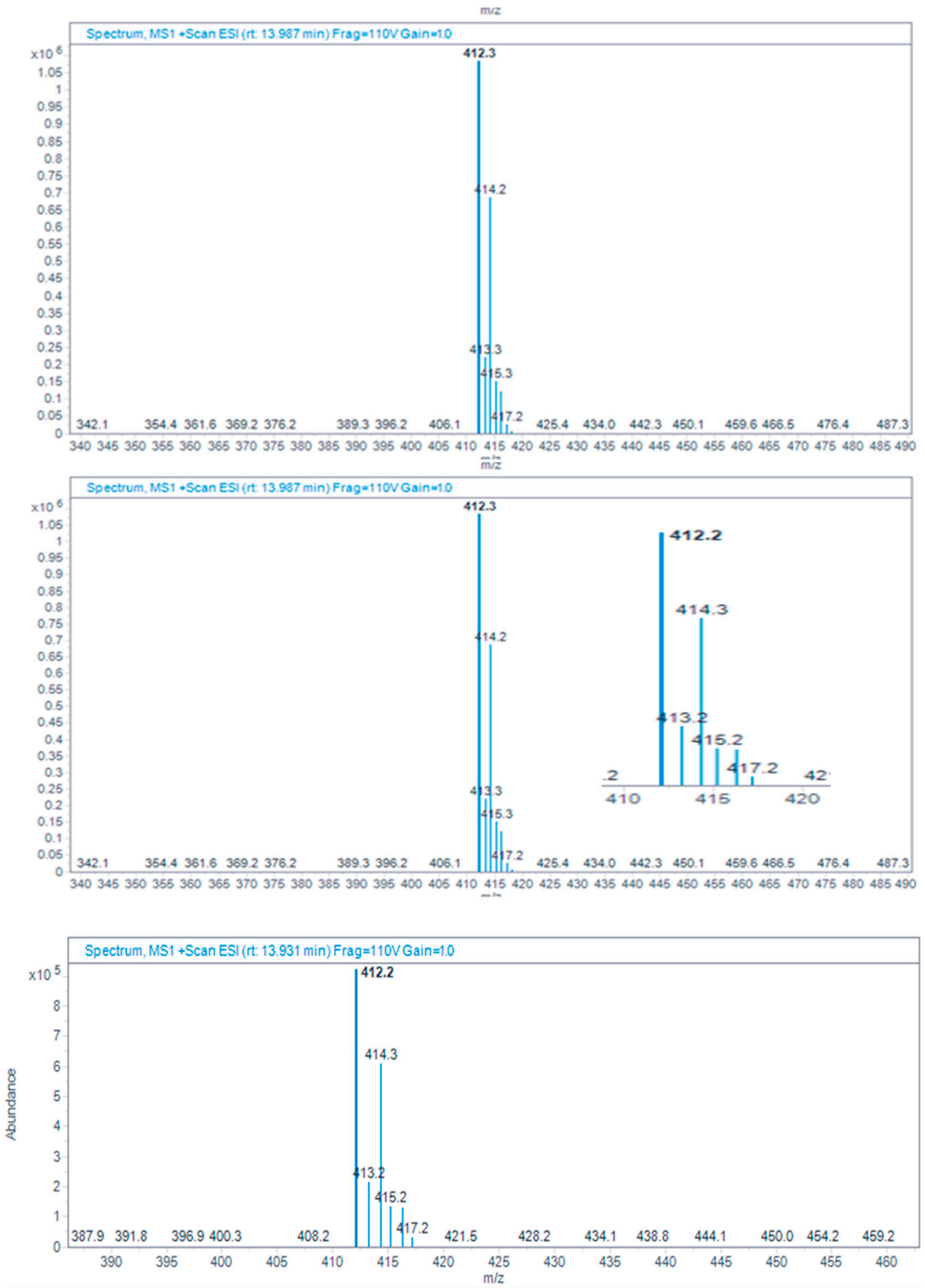

Figure S3: $[M+H]^+$ 412 for sample **PB1**

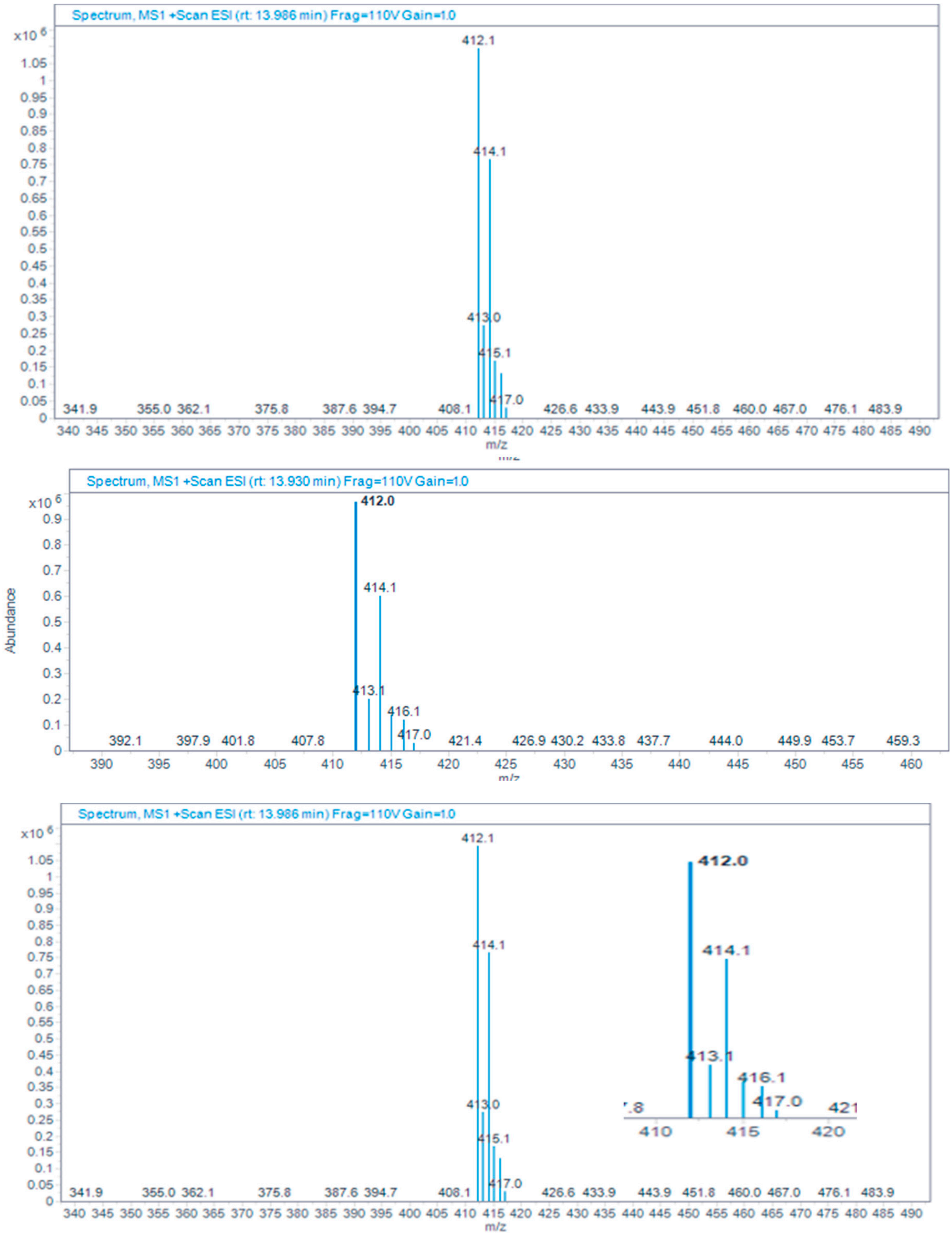

Figure S4: $[M+H]^+$ 412 for samples PB1 for sample **IQS016**

1.2. X-ray powder diffraction analysis of IQS016 and PB1

Table S1: Peak list for sample **PB1** from 2 to 40 º2θ

Pos. [°2θ]	d-spacing [Å]	Rel. Int. [%]
10.0761	8.77161	100
10.7302	8.23833	1.71
12.3275	7.17422	33.65
12.8616	6.87746	28.66
13.8554	6.38633	18.12
14.167	6.24656	28.51
14.9567	5.9185	2.57
15.5835	5.68179	3.1
15.7867	5.60913	37.38
17.0758	5.18846	2.58
17.4348	5.08243	9.92
17.8266	4.97161	26.54
17.9203	4.94581	60.22
19.0179	4.66277	8.35
19.285	4.5988	3.79
19.6858	4.50606	6.91
19.8828	4.46185	5.35
20.1457	4.40421	75.83
20.7828	4.27063	32.32
21.3759	4.15345	10.8
22.0603	4.02611	10.84
22.5824	3.93421	11.41
22.9308	3.87521	20.67
23.6574	3.7578	3.61
23.812	3.73376	6.78
23.9518	3.71227	4.92
24.1507	3.68216	4.89
24.358	3.65128	9.01
24.839	3.58166	11.02
25.3146	3.51543	65.01
25.9257	3.43394	10.98
26.2599	3.39099	3.74
26.6511	3.34209	29.95
27.1966	3.27629	2.48
27.7347	3.21393	5.31
27.9624	3.18827	8.53
28.3438	3.14623	1.36
28.7578	3.10187	1.16
29.2093	3.05495	9.24
29.6226	3.01326	2.28
30.0459	2.97176	9.44
30.8061	2.90014	9.25
31.4898	2.83872	3.54
31.9329	2.80033	3.21
32.101	2.78605	3.49
32.3319	2.76667	3.73
32.6446	2.74088	3.85
32.8948	2.72061	1.43
33.45	2.67671	1.97
33.7528	2.65339	1.18
34.2714	2.61441	3.27
34.8059	2.57548	10.43
35.4481	2.53028	1.53
35.7808	2.50751	2.89
36.6592	2.44942	4.92
37.5627	2.39255	1.8
37.6836	2.38515	2.44
37.9321	2.37009	2.52
38.7396	2.32253	2.59
38.9848	2.30848	1.79
39.3351	2.28873	2.26
39.932	2.25588	1.94

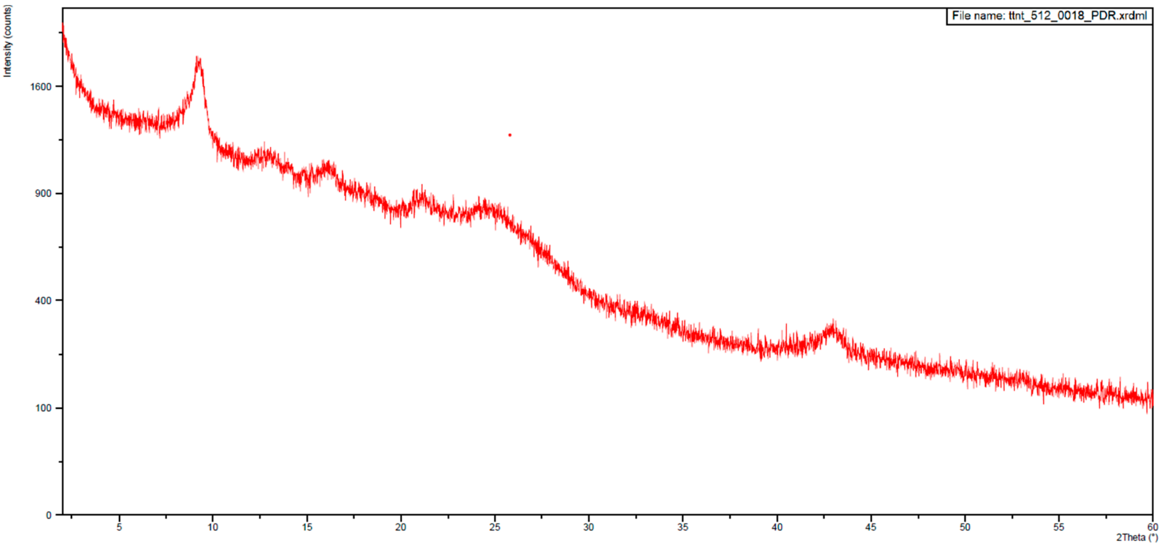

Figure S5: X-ray powder diffraction diagram of sample **IQS016**.

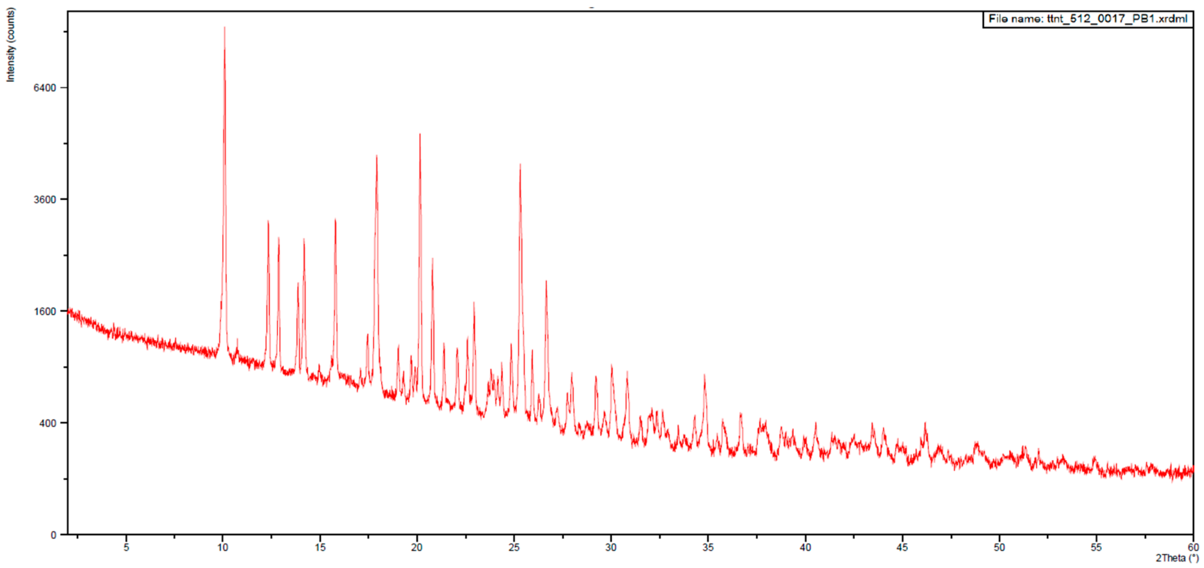

Figure S6: X-ray powder diffraction diagram of sample **PB1**.

1.3. Determination of the crystal structure of PB1 from X-ray powder diffraction data

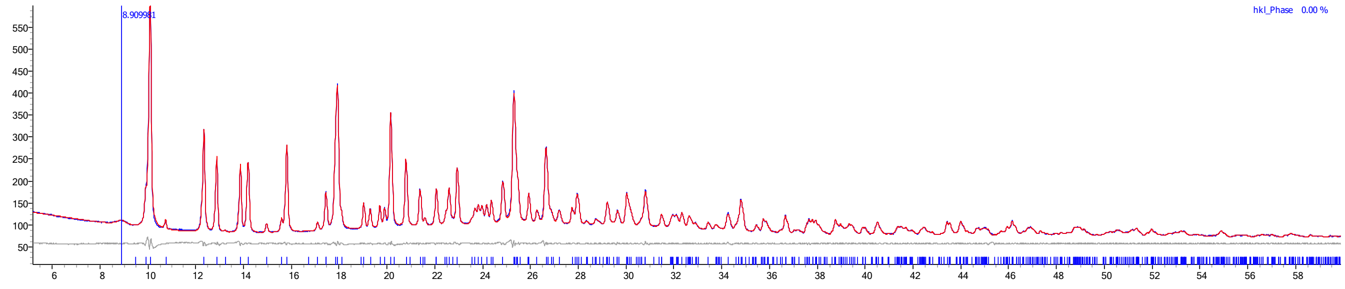

Figure S7: Pattern matching Pawley fit plot of **1**; agreement factor: Rwp = 2.63%. The plot shows the experimental PXRD profile (blue solid line), the calculated PXRD profile (red solid line) and the difference profile (grey, lower line). Blue tick marks indicate the peak positions.

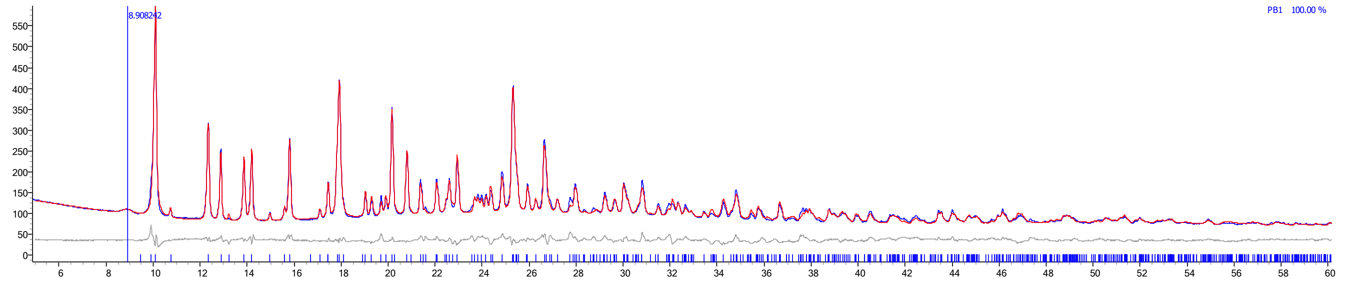

Figure S8: Final Rietveld plot for the crystal structure refinement of **1**; agreement factors: Rwp = 6.59% and Rp = 4.77%. The plot shows the experimental PXRD profile (blue solid line), the calculated PXRD profile (red solid line) and the difference profile (grey, lower line). Blue tick marks indicate the peak positions.

Table S2 summarizes the most relevant parameters of the crystal structure determination and refinement of **PB1**.

Table S2: Crystal data and structure refinement parameters of **PB1** from powder X-ray diffraction

Empirical formula	$C_{20}H_{15}Cl_2N_5O$
Formula Weight	412.27
Temperature (K)	298
Wavelength (Å)	1.54180
Crystal system	Orthorhombic
Space group	*Pbca*
a (Å)	18.6367(6)
b (Å)	17.4982(5)
c (Å)	12.4676(3)
α (°)	90
β (°)	90
γ (°)	90
Volume ($Å^3$)	4065.80(19)
Z, Z'	8, 1
Density (calc.) (Mg/m^3)	1.347
Measured 2θ range	2.018 to 69.9820
Stepsize (°)	0.013
Measured data points	5230
Rietveld Refinement Details:	
Profile function	Double-Voigt
2θ range used (°)	5.0 to 69.98
Num. of reflections	889
Data points	5000
Parameters	94
R_{wp}	6.59
R_p	4.77
R_{Bragg}	3.26
GoF	7.15

1.4. Crystal structure determination of a single crystal of IQS016 grown in MeOH

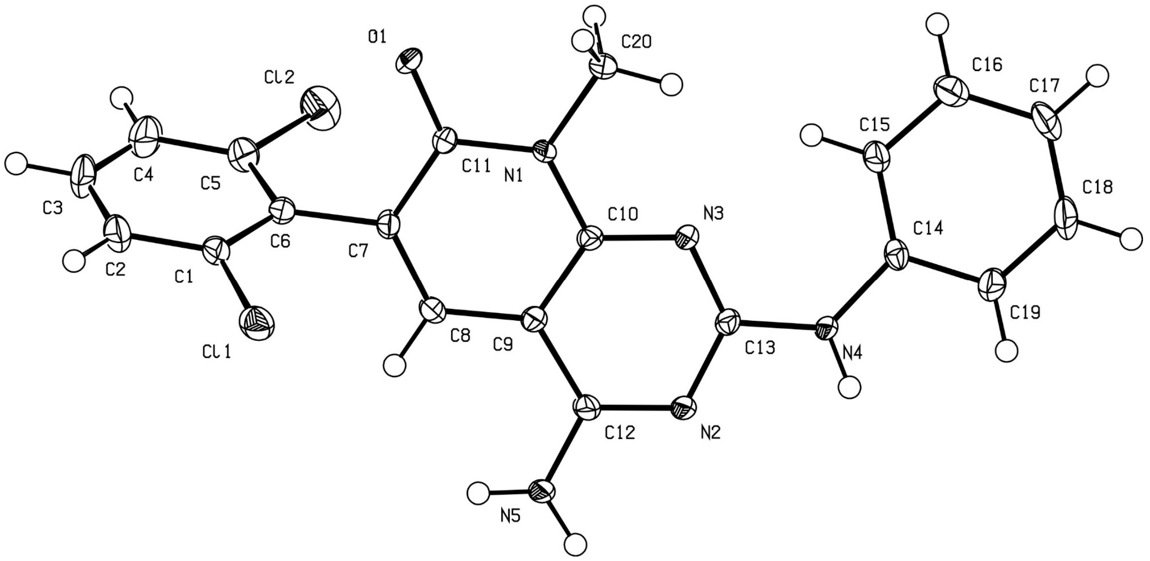

Figure S9: ORTEP diagram and atomic numbering of **1**.

Table S3: Crystal data and structure refinement for **1** (mo_023VB113_0ma_a).

Identification code	mo_023VB113_0ma_a	
Empirical formula	C20 H15 Cl2 N5 O	
Formula weight	412.27	
Temperature	100(2) K	
Wavelength	0.71073 Å	
Crystal system	Orthorhombic	
Space group	P b c a	
Unit cell dimensions	a = 16.9088(6) Å	α= 90°.
	b = 12.6026(4) Å	β= 90°.
	c = 18.5117(5) Å	γ = 90°.
Volume	3944.7(2) $Å^3$	
Z	8	
Density (calculated)	1.388 Mg/m^3	
Absorption coefficient	0.350 mm^{-1}	
F(000)	1696	
Crystal size	0.413 x 0.129 x 0.104 mm^3	
Theta range for data collection	2.296 to 26.398°.	
Index ranges	-21<=h<=21, -15<=k<=15, -23<=l<=22	
Reflections collected	79253	
Independent reflections	4037 [R(int) = 0.0516]	
Completeness to theta = 25.242°	99.8 %	
Absorption correction	Semi-empirical from equivalents	
Max. and min. transmission	0.7454 and 0.7161	
Refinement method	Full-matrix least-squares on F^2	
Data / restraints / parameters	4037 / 0 / 253	
Goodness-of-fit on F^2	1.149	
Final R indices [I>2sigma(I)]	R1 = 0.0463, wR2 = 0.1057	
R indices (all data)	R1 = 0.0530, wR2 = 0.1094	
Extinction coefficient	n/a	
Largest diff. peak and hole	0.424 and -0.448 e.$Å^{-3}$	
CCDC	2325664	

Table S4: Atomic coordinates (x 10^4) and equivalent isotropic displacement parameters ($Å^2$x 10^3) for **1** (mo_023VB113_0ma_a). U(eq) is defined as one third of the trace of the orthogonalized U^{ij} tensor.

	x	y	z	U(eq)
Cl(1)	5193(1)	417(1)	1351(1)	25(1)
Cl(2)	3897(1)	4240(1)	2002(1)	32(1)
O(1)	5829(1)	3749(1)	1872(1)	15(1)
N(1)	5984(1)	3256(1)	3043(1)	13(1)
N(2)	5499(1)	1332(2)	4772(1)	19(1)
N(3)	6185(1)	2811(1)	4244(1)	14(1)
N(4)	6330(1)	2334(1)	5451(1)	16(1)
N(5)	4628(2)	326(2)	4124(1)	32(1)
C(1)	4612(1)	1512(2)	1130(1)	21(1)
C(2)	4230(2)	1511(2)	464(1)	32(1)
C(3)	3764(2)	2367(3)	279(2)	38(1)
C(4)	3667(2)	3209(2)	746(2)	35(1)
C(5)	4054(1)	3193(2)	1405(1)	24(1)
C(6)	4550(1)	2356(2)	1613(1)	18(1)
C(7)	4986(1)	2391(2)	2312(1)	15(1)
C(8)	4827(1)	1732(2)	2872(1)	18(1)
C(9)	5232(1)	1822(2)	3537(1)	17(1)
C(10)	5798(1)	2616(2)	3625(1)	14(1)
C(11)	5610(1)	3172(2)	2377(1)	12(1)
C(12)	5118(2)	1148(2)	4150(1)	20(1)
C(13)	5992(1)	2172(2)	4789(1)	14(1)
C(14)	6806(1)	3179(2)	5699(1)	15(1)
C(15)	6929(1)	4124(2)	5326(1)	19(1)
C(16)	7363(2)	4930(2)	5643(1)	27(1)
C(17)	7667(2)	4821(3)	6331(2)	40(1)
C(18)	7555(2)	3879(3)	6695(2)	43(1)
C(19)	7134(2)	3054(2)	6383(1)	27(1)
C(20)	6608(1)	4060(2)	3123(1)	22(1)

Table S5: Bond lengths [Å] and angles [°] for **1 (**mo_023VB113_0ma_a).

Cl(1)-C(1)	1.742(3)
Cl(2)-C(5)	1.741(3)
O(1)-C(11)	1.242(3)
N(1)-C(10)	1.383(3)
N(1)-C(11)	1.388(3)
N(1)-C(20)	1.471(3)
N(2)-C(12)	1.340(3)
N(2)-C(13)	1.347(3)
N(3)-C(13)	1.332(3)
N(3)-C(10)	1.341(3)
N(4)-C(13)	1.369(3)
N(4)-C(14)	1.412(3)
N(4)-H(4N)	0.8800
N(5)-C(12)	1.327(3)
N(5)-H(5A)	0.8800
N(5)-H(5B)	0.8800
C(1)-C(2)	1.392(3)
C(1)-C(6)	1.392(3)
C(2)-C(3)	1.379(4)
C(2)-H(2A)	0.9500
C(3)-C(4)	1.378(4)
C(3)-H(3)	0.9500
C(4)-C(5)	1.386(4)
C(4)-H(4)	0.9500
C(5)-C(6)	1.402(3)
C(6)-C(7)	1.490(3)
C(7)-C(8)	1.354(3)
C(7)-C(11)	1.447(3)
C(8)-C(9)	1.414(3)
C(8)-H(8)	0.9500
C(9)-C(10)	1.395(3)
C(9)-C(12)	1.430(3)
C(14)-C(19)	1.391(3)
C(14)-C(15)	1.392(3)
C(15)-C(16)	1.383(3)
C(15)-H(15)	0.9500
C(16)-C(17)	1.381(4)

C(16)-H(16)	0.9500
C(17)-C(18)	1.379(4)
C(17)-H(17)	0.9500
C(18)-C(19)	1.386(4)
C(18)-H(18)	0.9500
C(19)-H(19)	0.9500
C(20)-H(20A)	0.9800
C(20)-H(20B)	0.9800
C(20)-H(20C)	0.9800

C(10)-N(1)-C(11)	122.96(18)
C(10)-N(1)-C(20)	119.09(17)
C(11)-N(1)-C(20)	117.94(17)
C(12)-N(2)-C(13)	116.94(19)
C(13)-N(3)-C(10)	114.59(18)
C(13)-N(4)-C(14)	129.97(19)
C(13)-N(4)-H(4N)	115.0
C(14)-N(4)-H(4N)	115.0
C(12)-N(5)-H(5A)	120.0
C(12)-N(5)-H(5B)	120.0
H(5A)-N(5)-H(5B)	120.0
C(2)-C(1)-C(6)	122.3(2)
C(2)-C(1)-Cl(1)	117.9(2)
C(6)-C(1)-Cl(1)	119.78(18)
C(3)-C(2)-C(1)	119.0(3)
C(3)-C(2)-H(2A)	120.5
C(1)-C(2)-H(2A)	120.5
C(4)-C(3)-C(2)	121.0(2)
C(4)-C(3)-H(3)	119.5
C(2)-C(3)-H(3)	119.5
C(3)-C(4)-C(5)	119.0(3)
C(3)-C(4)-H(4)	120.5
C(5)-C(4)-H(4)	120.5
C(4)-C(5)-C(6)	122.4(2)
C(4)-C(5)-Cl(2)	118.4(2)
C(6)-C(5)-Cl(2)	119.23(18)
C(1)-C(6)-C(5)	116.3(2)
C(1)-C(6)-C(7)	122.9(2)
C(5)-C(6)-C(7)	120.7(2)

C(8)-C(7)-C(11)	119.83(19)
C(8)-C(7)-C(6)	123.19(19)
C(11)-C(7)-C(6)	116.98(18)
C(7)-C(8)-C(9)	121.4(2)
C(7)-C(8)-H(8)	119.3
C(9)-C(8)-H(8)	119.3
C(10)-C(9)-C(8)	119.39(19)
C(10)-C(9)-C(12)	115.2(2)
C(8)-C(9)-C(12)	125.4(2)
N(3)-C(10)-N(1)	116.62(18)
N(3)-C(10)-C(9)	124.47(19)
N(1)-C(10)-C(9)	118.92(19)
O(1)-C(11)-N(1)	119.21(19)
O(1)-C(11)-C(7)	123.55(19)
N(1)-C(11)-C(7)	117.24(18)
N(5)-C(12)-N(2)	117.8(2)
N(5)-C(12)-C(9)	121.2(2)
N(2)-C(12)-C(9)	120.9(2)
N(3)-C(13)-N(2)	127.6(2)
N(3)-C(13)-N(4)	119.10(19)
N(2)-C(13)-N(4)	113.32(19)
C(19)-C(14)-C(15)	119.3(2)
C(19)-C(14)-N(4)	115.9(2)
C(15)-C(14)-N(4)	124.70(19)
C(16)-C(15)-C(14)	119.8(2)
C(16)-C(15)-H(15)	120.1
C(14)-C(15)-H(15)	120.1
C(17)-C(16)-C(15)	121.0(2)
C(17)-C(16)-H(16)	119.5
C(15)-C(16)-H(16)	119.5
C(18)-C(17)-C(16)	119.0(2)
C(18)-C(17)-H(17)	120.5
C(16)-C(17)-H(17)	120.5
C(17)-C(18)-C(19)	120.9(2)
C(17)-C(18)-H(18)	119.6
C(19)-C(18)-H(18)	119.6
C(18)-C(19)-C(14)	119.9(2)
C(18)-C(19)-H(19)	120.1
C(14)-C(19)-H(19)	120.1

N(1)-C(20)-H(20A)	109.5
N(1)-C(20)-H(20B)	109.5
H(20A)-C(20)-H(20B)	109.5
N(1)-C(20)-H(20C)	109.5
H(20A)-C(20)-H(20C)	109.5
H(20B)-C(20)-H(20C)	109.5

Table S6: Anisotropic displacement parameters ($Å^2 x 10^3$) for **1** (mo_023VB113_0ma_a). The anisotropic displacement factor exponent takes the form: $-2\pi^2[h^2 a^{*2}U^{11} + ... + 2 h k a^* b^* U^{12}]$

	U^{11}	U^{22}	U^{33}	U^{23}	U^{13}	U^{12}
Cl(1)	31(1)	23(1)	22(1)	-4(1)	3(1)	-5(1)
Cl(2)	24(1)	31(1)	42(1)	-4(1)	-6(1)	6(1)
O(1)	18(1)	16(1)	11(1)	4(1)	-1(1)	0(1)
N(1)	14(1)	14(1)	12(1)	1(1)	-1(1)	-6(1)
N(2)	32(1)	14(1)	10(1)	1(1)	-1(1)	-9(1)
N(3)	18(1)	14(1)	10(1)	1(1)	-1(1)	-3(1)
N(4)	26(1)	12(1)	9(1)	1(1)	-2(1)	-5(1)
N(5)	59(2)	27(1)	10(1)	4(1)	-5(1)	-28(1)
C(1)	22(1)	25(1)	16(1)	2(1)	-1(1)	-10(1)
C(2)	39(2)	38(2)	17(1)	-2(1)	-5(1)	-17(1)
C(3)	38(2)	52(2)	23(1)	7(1)	-18(1)	-14(1)
C(4)	30(1)	42(2)	32(2)	10(1)	-15(1)	-4(1)
C(5)	21(1)	28(1)	24(1)	2(1)	-5(1)	-4(1)
C(6)	16(1)	23(1)	14(1)	3(1)	-1(1)	-8(1)
C(7)	15(1)	19(1)	11(1)	-1(1)	-1(1)	-2(1)
C(8)	22(1)	18(1)	14(1)	-1(1)	0(1)	-9(1)
C(9)	23(1)	16(1)	11(1)	0(1)	1(1)	-5(1)
C(10)	16(1)	13(1)	12(1)	0(1)	2(1)	-1(1)
C(11)	12(1)	14(1)	11(1)	0(1)	1(1)	1(1)
C(12)	31(1)	17(1)	12(1)	-2(1)	1(1)	-10(1)
C(13)	21(1)	12(1)	10(1)	-1(1)	0(1)	1(1)
C(14)	15(1)	19(1)	12(1)	-4(1)	0(1)	-1(1)
C(15)	22(1)	20(1)	15(1)	-3(1)	-1(1)	-4(1)
C(16)	31(1)	25(1)	25(1)	-1(1)	1(1)	-11(1)
C(17)	47(2)	47(2)	27(1)	-2(1)	-10(1)	-32(2)
C(18)	48(2)	60(2)	22(1)	6(1)	-19(1)	-28(2)
C(19)	26(1)	36(1)	20(1)	8(1)	-8(1)	-12(1)
C(20)	22(1)	26(1)	16(1)	5(1)	-2(1)	-14(1)

Table S7: Hydrogen coordinates (x 10^4) and isotropic displacement parameters ($Å^2$x 10^{3}) for **1** (mo_023VB113_0ma_a).

	x	y	z	U(eq)
H(4N)	6236	1835	5772	19
H(5A)	4562	-77	4508	38
H(5B)	4369	183	3723	38
H(2A)	4289	929	142	38
H(3)	3506	2376	-177	45
H(4)	3340	3792	617	42
H(8)	4435	1199	2815	22
H(15)	6716	4215	4855	23
H(16)	7452	5569	5383	32
H(17)	7951	5387	6550	48
H(18)	7769	3794	7166	52
H(19)	7069	2404	6637	32
H(20A)	7018	3941	2758	32
H(20B)	6841	4007	3606	32
H(20C)	6380	4769	3057	32

Table S8: Analysis of Potential Hydrogen Bond for **1** (mo_023VB113_0ma_a).

Donor --- H....Acceptor	[ARU]	D - H	H...A	D...A	D - H...A
N(4) --H(4N) ..O(1)	[x,1/2-y,-1/2+z]	0.88	2.27	3.0818	153
N(5) --H(5A) ..N(2)	[-x,-y,-z]	0.88	2.07	2.9299	165
N(5) --H(5B) ..O(1)	[-x,-1/2+y,1/2-z]	0.88	2.14	2.8181	133

1.5. Crystal structure determination of a single crystal of IQS016 grown in DMSO

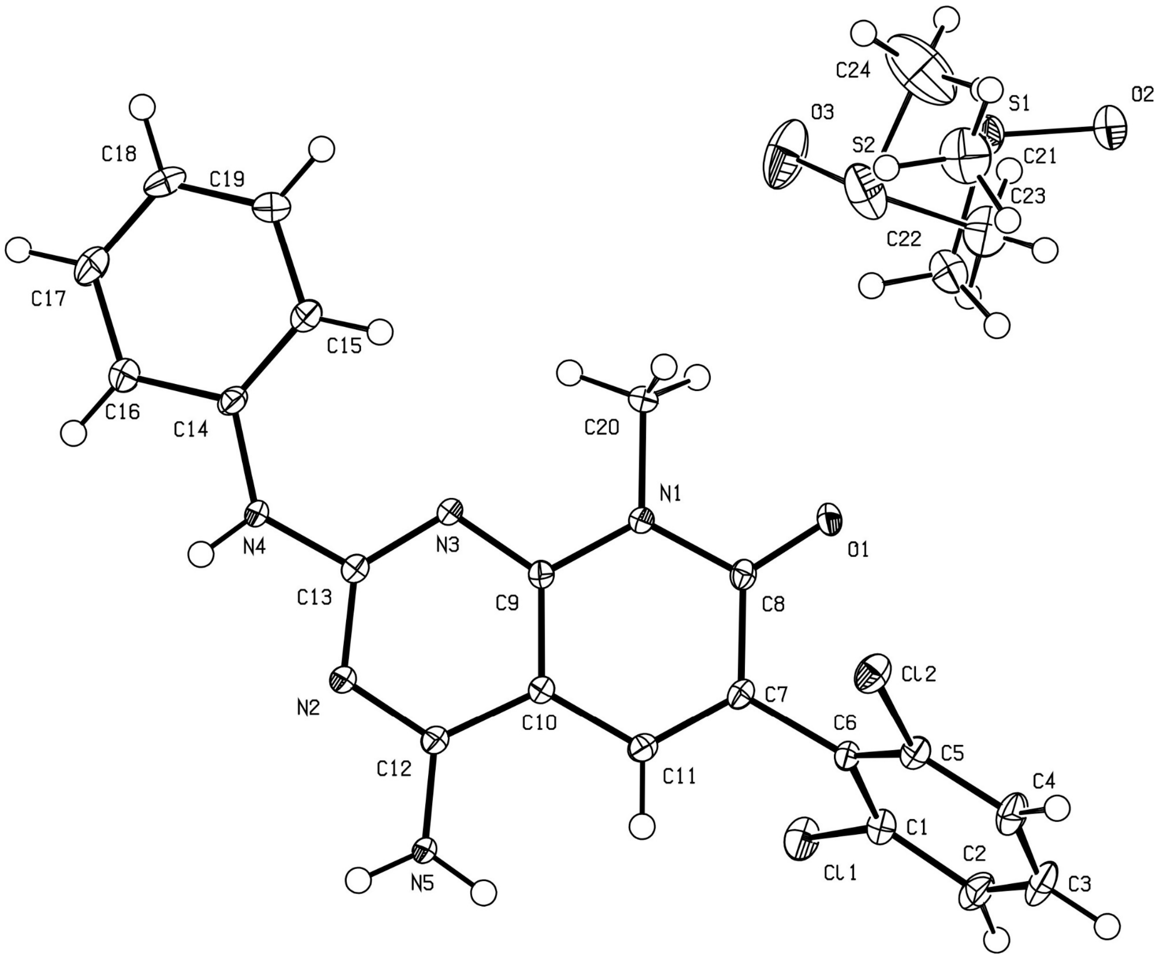

Figure S10: ORTEP diagram and atomic numbering of a DMSO solvate of **1**.

Table S9: Crystal data and structure refinement for a DMSO solvate of **1** (mo_023VB102_0m_a).

Identification code	mo_023VB102_0m_a	
Empirical formula	C22 H21 Cl2 N5 O2 S	
Formula weight	490.40	
Temperature	100(2) K	
Wavelength	0.71073 Å	
Crystal system	Monoclinic	
Space group	C 2/c	
Unit cell dimensions	a = 20.0528(9) Å	α= 90°.
	b = 11.8577(5) Å	β= 90.855(2)°.
	c = 18.8759(8) Å	γ = 90°.
Volume	4487.8(3) $Å^3$	
Z	8	
Density (calculated)	1.452 Mg/m^3	
Absorption coefficient	0.413 mm^{-1}	
F(000)	2032	
Crystal size	0.266 x 0.123 x 0.092 mm^3	
Theta range for data collection	1.995 to 27.147°.	
Index ranges	-25<=h<=25, -15<=k<=15, -24<=l<=21	
Reflections collected	34473	
Independent reflections	4959 [R(int) = 0.0369]	
Completeness to theta = 25.242°	99.7 %	
Absorption correction	Semi-empirical from equivalents	
Max. and min. transmission	0.7455 and 0.7026	
Refinement method	Full-matrix least-squares on F^2	
Data / restraints / parameters	4959 / 0 / 333	
Goodness-of-fit on F^2	1.067	
Final R indices [I>2sigma(I)]	R1 = 0.0325, wR2 = 0.0779	
R indices (all data)	R1 = 0.0425, wR2 = 0.0854	
Extinction coefficient	n/a	
Largest diff. peak and hole	0.396 and -0.322 e.$Å^{-3}$	
CCDC	2325663	

Table S10: Atomic coordinates (x 10^4) and equivalent isotropic displacement parameters ($Å^2$x 10^3) for a DMSO solvate of **1** (mo_023VB102_0m_a). U(eq) is defined as one third of the trace of the orthogonalized U^{ij} tensor.

	x	y	z	U(eq)
Cl(1)	7079(1)	7348(1)	6412(1)	22(1)
Cl(2)	8330(1)	3385(1)	6929(1)	20(1)
O(1)	6671(1)	3714(1)	6853(1)	14(1)
N(1)	6568(1)	4192(1)	8015(1)	12(1)
N(2)	7059(1)	6110(1)	9757(1)	14(1)
N(3)	6445(1)	4582(1)	9215(1)	13(1)
N(4)	6314(1)	5121(1)	10395(1)	16(1)
N(5)	7829(1)	7136(1)	9148(1)	18(1)
C(1)	7639(1)	6295(1)	6181(1)	16(1)
C(2)	8012(1)	6454(2)	5573(1)	22(1)
C(3)	8460(1)	5635(2)	5374(1)	25(1)
C(4)	8546(1)	4669(2)	5776(1)	22(1)
C(5)	8177(1)	4548(1)	6388(1)	16(1)
C(6)	7711(1)	5340(1)	6610(1)	13(1)
C(7)	7358(1)	5200(1)	7292(1)	12(1)
C(8)	6857(1)	4328(1)	7353(1)	12(1)
C(9)	6748(1)	4824(1)	8605(1)	12(1)
C(10)	7226(1)	5670(1)	8528(1)	12(1)
C(11)	7525(1)	5843(1)	7864(1)	13(1)
C(12)	7372(1)	6315(1)	9152(1)	13(1)
C(13)	6612(1)	5257(1)	9757(1)	13(1)
C(14)	5788(1)	4408(1)	10600(1)	14(1)
C(15)	5623(1)	3402(1)	10265(1)	22(1)
C(16)	5444(1)	4734(1)	11206(1)	20(1)
C(17)	4940(1)	4062(2)	11466(1)	28(1)
C(18)	4776(1)	3061(2)	11133(1)	33(1)
C(19)	5120(1)	2734(2)	10538(1)	29(1)
C(20)	6050(1)	3319(1)	8077(1)	19(1)
S(1)	9860(1)	4472(1)	3879(1)	22(1)
O(2)	9857(1)	4793(2)	4651(1)	23(1)
C(21)	9145(2)	5141(4)	3472(2)	36(1)
C(22)	9545(2)	3061(4)	3834(2)	29(1)
S(2)	9462(1)	4074(1)	3061(1)	35(1)

O(3)	10059(2)	3607(3)	2720(1)	45(1)
C(23)	9533(3)	3663(6)	3966(3)	55(2)
C(24)	9614(3)	5542(4)	3180(3)	65(2)

Table S11: Bond lengths [Å] and angles [°] for a DMSO solvate of **1** (mo_023VB102_0m_a).

Cl(1)-C(1)	1.7401(16)
Cl(2)-C(5)	1.7403(16)
O(1)-C(8)	1.2458(18)
N(1)-C(9)	1.3852(18)
N(1)-C(8)	1.3950(18)
N(1)-C(20)	1.4716(18)
N(2)-C(12)	1.3337(18)
N(2)-C(13)	1.3499(19)
N(3)-C(13)	1.3368(19)
N(3)-C(9)	1.3417(18)
N(4)-C(13)	1.3635(18)
N(4)-C(14)	1.4099(19)
N(4)-H(4N)	0.84(2)
N(5)-C(12)	1.3377(19)
N(5)-H(5A)	0.8800
N(5)-H(5B)	0.8800
C(1)-C(2)	1.392(2)
C(1)-C(6)	1.398(2)
C(2)-C(3)	1.378(2)
C(2)-H(2)	0.9500
C(3)-C(4)	1.383(2)
C(3)-H(3)	0.9500
C(4)-C(5)	1.387(2)
C(4)-H(4)	0.9500
C(5)-C(6)	1.395(2)
C(6)-C(7)	1.4885(19)
C(7)-C(11)	1.359(2)
C(7)-C(8)	1.447(2)
C(9)-C(10)	1.396(2)
C(10)-C(11)	1.4130(19)
C(10)-C(12)	1.431(2)
C(11)-H(11)	0.9500
C(14)-C(15)	1.387(2)
C(14)-C(16)	1.398(2)
C(15)-C(19)	1.388(2)
C(15)-H(15)	0.9500
C(16)-C(17)	1.383(2)

C(16)-H(16)	0.9500
C(17)-C(18)	1.380(3)
C(17)-H(17)	0.9500
C(18)-C(19)	1.384(3)
C(18)-H(18)	0.9500
C(19)-H(19)	0.9500
C(20)-H(20A)	0.9800
C(20)-H(20B)	0.9800
C(20)-H(20C)	0.9800
S(1)-O(2)	1.506(2)
S(1)-C(22)	1.790(5)
S(1)-C(21)	1.801(4)
C(21)-H(21A)	0.9800
C(21)-H(21B)	0.9800
C(21)-H(21C)	0.9800
C(22)-H(22A)	0.9800
C(22)-H(22B)	0.9800
C(22)-H(22C)	0.9800
S(2)-O(3)	1.475(3)
S(2)-C(23)	1.779(6)
S(2)-C(24)	1.781(5)
C(23)-H(23A)	0.9800
C(23)-H(23B)	0.9800
C(23)-H(23C)	0.9800
C(24)-H(24A)	0.9800
C(24)-H(24B)	0.9800
C(24)-H(24C)	0.9800
C(9)-N(1)-C(8)	123.36(12)
C(9)-N(1)-C(20)	119.53(12)
C(8)-N(1)-C(20)	117.11(12)
C(12)-N(2)-C(13)	117.22(13)
C(13)-N(3)-C(9)	114.66(12)
C(13)-N(4)-C(14)	130.80(13)
C(13)-N(4)-H(4N)	114.6(13)
C(14)-N(4)-H(4N)	114.3(13)
C(12)-N(5)-H(5A)	120.0
C(12)-N(5)-H(5B)	120.0
H(5A)-N(5)-H(5B)	120.0

C(2)-C(1)-C(6)	122.31(14)
C(2)-C(1)-Cl(1)	117.81(12)
C(6)-C(1)-Cl(1)	119.86(11)
C(3)-C(2)-C(1)	119.32(15)
C(3)-C(2)-H(2)	120.3
C(1)-C(2)-H(2)	120.3
C(2)-C(3)-C(4)	120.66(15)
C(2)-C(3)-H(3)	119.7
C(4)-C(3)-H(3)	119.7
C(3)-C(4)-C(5)	118.61(15)
C(3)-C(4)-H(4)	120.7
C(5)-C(4)-H(4)	120.7
C(4)-C(5)-C(6)	123.23(15)
C(4)-C(5)-Cl(2)	118.67(12)
C(6)-C(5)-Cl(2)	118.02(11)
C(5)-C(6)-C(1)	115.84(13)
C(5)-C(6)-C(7)	120.94(13)
C(1)-C(6)-C(7)	123.05(13)
C(11)-C(7)-C(8)	120.06(13)
C(11)-C(7)-C(6)	120.62(13)
C(8)-C(7)-C(6)	119.25(13)
O(1)-C(8)-N(1)	119.18(13)
O(1)-C(8)-C(7)	123.91(13)
N(1)-C(8)-C(7)	116.91(12)
N(3)-C(9)-N(1)	117.28(13)
N(3)-C(9)-C(10)	124.25(13)
N(1)-C(9)-C(10)	118.47(12)
C(9)-C(10)-C(11)	119.83(13)
C(9)-C(10)-C(12)	115.52(12)
C(11)-C(10)-C(12)	124.65(13)
C(7)-C(11)-C(10)	121.34(13)
C(7)-C(11)-H(11)	119.3
C(10)-C(11)-H(11)	119.3
N(2)-C(12)-N(5)	117.99(13)
N(2)-C(12)-C(10)	120.91(13)
N(5)-C(12)-C(10)	121.10(13)
N(3)-C(13)-N(2)	127.36(13)
N(3)-C(13)-N(4)	119.77(13)
N(2)-C(13)-N(4)	112.86(13)

C(15)-C(14)-C(16)	119.55(14)
C(15)-C(14)-N(4)	124.37(14)
C(16)-C(14)-N(4)	116.00(14)
C(14)-C(15)-C(19)	119.43(15)
C(14)-C(15)-H(15)	120.3
C(19)-C(15)-H(15)	120.3
C(17)-C(16)-C(14)	120.20(15)
C(17)-C(16)-H(16)	119.9
C(14)-C(16)-H(16)	119.9
C(18)-C(17)-C(16)	120.34(16)
C(18)-C(17)-H(17)	119.8
C(16)-C(17)-H(17)	119.8
C(17)-C(18)-C(19)	119.43(16)
C(17)-C(18)-H(18)	120.3
C(19)-C(18)-H(18)	120.3
C(18)-C(19)-C(15)	121.04(17)
C(18)-C(19)-H(19)	119.5
C(15)-C(19)-H(19)	119.5
N(1)-C(20)-H(20A)	109.5
N(1)-C(20)-H(20B)	109.5
H(20A)-C(20)-H(20B)	109.5
N(1)-C(20)-H(20C)	109.5
H(20A)-C(20)-H(20C)	109.5
H(20B)-C(20)-H(20C)	109.5
O(2)-S(1)-C(22)	106.00(18)
O(2)-S(1)-C(21)	106.70(17)
C(22)-S(1)-C(21)	96.6(2)
S(1)-C(21)-H(21A)	109.5
S(1)-C(21)-H(21B)	109.5
H(21A)-C(21)-H(21B)	109.5
S(1)-C(21)-H(21C)	109.5
H(21A)-C(21)-H(21C)	109.5
H(21B)-C(21)-H(21C)	109.5
S(1)-C(22)-H(22A)	109.5
S(1)-C(22)-H(22B)	109.5
H(22A)-C(22)-H(22B)	109.5
S(1)-C(22)-H(22C)	109.5
H(22A)-C(22)-H(22C)	109.5
H(22B)-C(22)-H(22C)	109.5

O(3)-S(2)-C(23)	105.2(2)
O(3)-S(2)-C(24)	106.5(3)
C(23)-S(2)-C(24)	97.8(3)
C(23)-S(2)-O(3)#1	130.2(3)
C(24)-S(2)-O(3)#1	107.6(3)
S(2)-O(3)-S(2)#1	131.88(19)
S(2)-C(23)-H(23A)	109.5
S(2)-C(23)-H(23B)	109.5
H(23A)-C(23)-H(23B)	109.5
S(2)-C(23)-H(23C)	109.5
H(23A)-C(23)-H(23C)	109.5
H(23B)-C(23)-H(23C)	109.5
S(2)-C(24)-H(24A)	109.5
S(2)-C(24)-H(24B)	109.5
H(24A)-C(24)-H(24B)	109.5
S(2)-C(24)-H(24C)	109.5
H(24A)-C(24)-H(24C)	109.5
H(24B)-C(24)-H(24C)	109.5

Symmetry transformations used to generate equivalent atoms:

#1 -x+2,y,-z+1/2

Table S12: Anisotropic displacement parameters ($Å^2$x 10^3) for a DMSO solvate of **1** (mo_023VB102_0m_a). The anisotropic displacement factor exponent takes the form: $-2\pi^2[h^2 a^{*2}U^{11} + ... + 2 h k a^* b^* U^{12}]$

	U^{11}	U^{22}	U^{33}	U^{23}	U^{13}	U^{12}
Cl(1)	29(1)	19(1)	19(1)	3(1)	4(1)	6(1)
Cl(2)	19(1)	19(1)	21(1)	3(1)	6(1)	6(1)
O(1)	16(1)	15(1)	11(1)	-4(1)	2(1)	0(1)
N(1)	13(1)	13(1)	11(1)	-2(1)	3(1)	-3(1)
N(2)	17(1)	15(1)	10(1)	0(1)	2(1)	-5(1)
N(3)	15(1)	14(1)	11(1)	-1(1)	4(1)	-4(1)
N(4)	21(1)	18(1)	9(1)	-2(1)	4(1)	-8(1)
N(5)	24(1)	21(1)	9(1)	-2(1)	4(1)	-12(1)
C(1)	17(1)	19(1)	13(1)	-1(1)	2(1)	1(1)
C(2)	24(1)	27(1)	15(1)	7(1)	4(1)	-2(1)
C(3)	24(1)	38(1)	15(1)	3(1)	9(1)	1(1)
C(4)	19(1)	30(1)	18(1)	-2(1)	8(1)	3(1)
C(5)	15(1)	18(1)	13(1)	1(1)	2(1)	0(1)
C(6)	13(1)	17(1)	10(1)	-2(1)	2(1)	-2(1)
C(7)	12(1)	14(1)	11(1)	1(1)	3(1)	1(1)
C(8)	12(1)	12(1)	11(1)	-1(1)	2(1)	3(1)
C(9)	13(1)	12(1)	11(1)	-1(1)	1(1)	0(1)
C(10)	13(1)	12(1)	11(1)	0(1)	2(1)	-1(1)
C(11)	13(1)	13(1)	13(1)	1(1)	2(1)	0(1)
C(12)	14(1)	15(1)	11(1)	1(1)	1(1)	-2(1)
C(13)	14(1)	14(1)	11(1)	1(1)	2(1)	-1(1)
C(14)	15(1)	17(1)	12(1)	3(1)	2(1)	-2(1)
C(15)	29(1)	20(1)	17(1)	-1(1)	9(1)	-7(1)
C(16)	20(1)	23(1)	18(1)	-4(1)	6(1)	-4(1)
C(17)	24(1)	39(1)	23(1)	-5(1)	13(1)	-8(1)
C(18)	32(1)	40(1)	28(1)	-2(1)	13(1)	-21(1)
C(19)	39(1)	25(1)	24(1)	-3(1)	8(1)	-17(1)
C(20)	21(1)	20(1)	15(1)	-4(1)	4(1)	-10(1)
S(1)	18(1)	29(1)	18(1)	-2(1)	1(1)	1(1)
O(2)	26(1)	25(1)	18(1)	-4(1)	1(1)	4(1)
C(21)	30(2)	45(2)	34(2)	4(2)	-12(2)	8(2)
C(22)	32(2)	30(2)	24(2)	-6(2)	-1(2)	-4(2)
S(2)	29(1)	42(1)	35(1)	-18(1)	-6(1)	7(1)
O(3)	52(2)	52(2)	32(2)	6(1)	17(2)	26(2)

C(23)	59(3)	70(4)	35(3)	-13(3)	13(2)	-26(3)
C(24)	72(4)	37(3)	84(4)	-20(3)	-31(3)	15(3)

Table S13: Hydrogen coordinates (x 10^4) and isotropic displacement parameters (Å^{2}x 10^3) for a DMSO solvate of **1** (mo_023VB102_0m_a).

	x	y	z	U(eq)
H(4N)	6442(9)	5572(16)	10709(10)	19
H(5A)	7915	7522	9537	22
H(5B)	8044	7290	8757	22
H(2)	7959	7120	5298	26
H(3)	8711	5734	4956	31
H(4)	8852	4102	5637	26
H(11)	7849	6421	7815	16
H(15)	5852	3174	9852	26
H(16)	5557	5419	11439	24
H(17)	4706	4291	11875	34
H(18)	4429	2600	11312	40
H(19)	5011	2041	10312	35
H(20A)	5760	3335	7655	28
H(20B)	5784	3464	8499	28
H(20C)	6262	2576	8119	28
H(21A)	9200	5962	3492	54
H(21B)	8742	4925	3726	54
H(21C)	9104	4900	2977	54
H(22A)	9464	2853	3338	43
H(22B)	9127	3014	4094	43
H(22C)	9872	2543	4047	43
H(23A)	9500	2840	4001	82
H(23B)	9174	4012	4235	82
H(23C)	9965	3910	4159	82
H(24A)	9610	5918	2717	97
H(24B)	10049	5650	3412	97
H(24C)	9265	5866	3475	97

Table S14: Hydrogen Bonds for a DMSO solvate of **1** (mo_023VB102_0m_a).

N(4)	--H(4N)	..O(1)	[x,-y,1/2+z]	0.84	2.36	3.1512	159
N(5)	--H(5A)	..N(2)	[1/2-x,-1/2-y,1-z]	0.88	2.10	2.9392	159
N(5)	--H(5B)	..O(1)	[1/2-x,-1/2+y,1/2-z]	0.88	2.13	2.8512	139
